# Enhanced Axial Resolution of Wide-Field Two-Photon Excitation Microscopy by Line Scanning Using a Digital Micromirror Device

**DOI:** 10.3390/mi8030085

**Published:** 2017-03-09

**Authors:** Jong Kang Park, Christopher J. Rowlands, Peter T. C. So

**Affiliations:** 1 Department of Biological Engineering, Massachusetts Institute of Technology, Cambridge, MA 02139, USA; jongpark@mit.edu; 2 Department of Chemical Engineering and Biotechnology, Cambridge University, Cambridge CB2 1TN, UK; cjr52@cam.ac.uk; 3 Laser Biomedical Research Center (LBRC), Massachusetts Institute of Technology, Cambridge, MA 02139, USA

**Keywords:** multiphoton microscopy, temporal focusing, digital micromirror device

## Abstract

Temporal focusing multiphoton microscopy is a technique for performing highly parallelized multiphoton microscopy while still maintaining depth discrimination. While the conventional wide-field configuration for temporal focusing suffers from sub-optimal axial resolution, line scanning temporal focusing, implemented here using a digital micromirror device (DMD), can provide substantial improvement. The DMD-based line scanning temporal focusing technique dynamically trades off the degree of parallelization, and hence imaging speed, for axial resolution, allowing performance parameters to be adapted to the experimental requirements. We demonstrate this new instrument in calibration specimens and in biological specimens, including a mouse kidney slice.

## 1. Introduction

Multiphoton-excited fluorescence microscopy is a technique that can obtain axially resolved fluorescence images of various specimens, and is often employed in biomedical applications on account of its high resolution, intrinsic optical sectioning capability, and biocompatibility [1]. Since its invention, and with the aid of recent advances in femtosecond pulsed lasers, it has now become one of the standard microscopic tools in the field of biology [2,3,4,5]. Despite its utility for structural and functional studies on biological systems, conventional multiphoton microscopy is slow, based as it is on conventional raster-scanning of a tightly focused laser beam; the maximum imaging speed is often limited by the physical speed of a scanning mirror. This mechanical limitation hampers studies of the high-speed dynamics of a system of interest. To illuminate large areas at high speeds, new methods are needed [6].

Temporal focusing, a highly parallelized excitation technique, was proposed independently by Oron et al. and Zhu et al. [7,8]. This technique achieves parallelization of the excitation process by reflecting a high-peak-power femtosecond optical pulse off of a diffraction grating, and imaging the light from the grating onto the sample of interest using a high-resolution microscope. By spatially dispersing the optical pulse with the grating, the laser’s pulse width is broadened temporally, and the peak power is reduced everywhere apart from at the focal plane of the microscope, thus ensuring that multiphoton excitation is confined to this plane. This technique has been successfully implemented in various applications including cellular dynamic imaging [9], depth-resolved lifetime imaging [10,11], optogenetics [12,13], and microfabrication [14,15,16]. Although this approach gives rise to wide-field multiphoton excitation, conventional wide-field temporal focusing does not achieve axial resolution comparable to point-scanning multiphoton microscopy, instead achieving axial resolution similar to that of a line-scanning multiphoton microscope [7,17].

Nevertheless, by patterning the temporal focusing excitation plane, the axial resolution can be improved. Line scanning temporal focusing is one example of patterning the excitation plane by focusing the light into a line, as demonstrated by Tal et al. [18]. In their paper, a cylindrical lens was used to generate a line pattern at the grating, and the resulting line shape at the sample plane had a ~1.5 µm axial extent, resulting in axial resolution close to that of conventional point-scanning multiphoton microscopy. This improved axial resolution does not come for free though; it requires the user to raster this excitation line along the orthogonal dimension using a galvo-mirror. 

In this paper, we demonstrate a simple and programmable implementation of the line scanning temporal focusing technique, by employing a digital micromirror device (DMD). Using the DMD, a number of lines can be illuminated simultaneously, and swept across the sample at high speed. Unlike in a conventional line-scanning temporal focusing system [18,19], this approach enables trivial modification of the number of lines and the line thickness, allowing the tradeoff between the illuminated area and the axial resolution to be explored. The number of simultaneously swept lines can be also increased arbitrarily, helping to increase the photon efficiency, since fewer photons will be lost as the fraction of “on” pixels on the DMD is increased. In this study, particular attention is paid to the axial resolution of this system as a function of line thickness and the number of lines, and to demonstrate that the technique is not limited to test samples, a sectioned mouse kidney slice was imaged to highlight the superior axial resolution of DMD-based line scanning temporal focusing compared to wide-field temporal focusing. The experimental results clearly show that the DMD-based line scanning temporal focusing technique, with its ability to control the parameters of the projected line patterns, provides improved axial resolution in biological microscopy while maintaining high pixel throughput.

## 2. Materials and Methods

### 2.1. Digital Micromirror Device (DMD)-Based Line Scanning Temporal Focusing Multi-Photon Microscope

**Figure 1:**
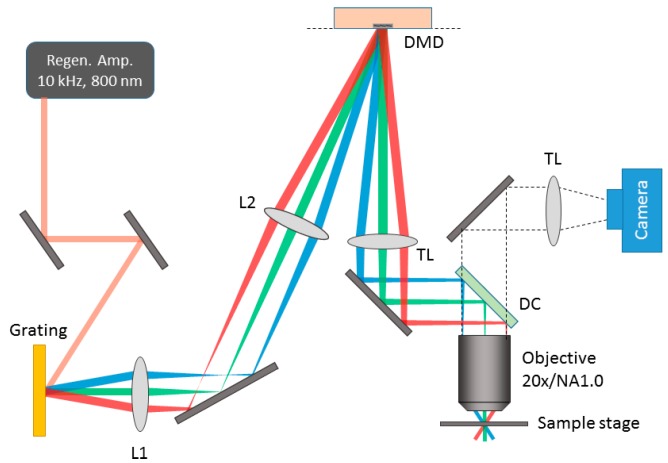
Schematic diagram of digital micromirror device (DMD)-based temporal focusing two-photon excited fluorescence microscope. L: lens; DMD: digital micromirror device; TL: tube lens; DC: dichroic mirror. L1: *f* = 300 mm, L2: *f* = 750 mm, TL (excitation): *f* = 750 mm.

Figure 1 shows the schematic diagram of the DMD-based line temporal focusing multiphoton microscope. An ultrafast pulsed laser beam (120 fs pulse width, 10 kHz repetition rate, ~8 mm beam diameter (1/e^2^)) from a regenerative amplifier (Legend Elite, Coherent, Santa Clara, CA, USA) was demagnified to ~6 mm and diffracted from a reflective ruled grating with a grating period of 1200 lines/mm (53033BK02-530R, Richardson Gratings, Rochester, NY, USA). The grating was followed by a 4f-lens system consisting of two planoconvex lenses (*f* = 300 mm; LA1256, Thorlabs and *f* = 750 mm; LA1727, Thorlabs, Newton, NJ, USA), which served to project and magnify the image of the grating onto the DMD (V-7000, Vialux, Chemnitz, Germany). The beam diameter of ~15 mm slightly overfilled the active area of the DMD. Arbitrary patterns could be uploaded onto the DMD using a control program (EasyProj) provided by Vialux. The light from the DMD was then allowed to pass through a tube lens (*f* = 750 mm; LA1227, Thorlabs), reflected off a dichroic filter (FF750-SDi02, Semrock, Rochester, NY, USA) and into an objective (water immersion 20×/1.0 numerical aperture; 421452-9880-000, Zeiss) to focus the image of the DMD into the sample. The geometric dispersion of the system ensured that the pulse width was broad enough to minimize multiphoton excitation except for at the sample plane. For wide-field temporal focusing, that is to say, when all 256 × 256 pixels of the DMD were on a field of view of ~60 µm × 60 µm was generated at the sample plane. The two-photon excited fluorescence from the sample was collected by the same objective lens and imaged by another tube lens (*f* = 200 mm; PAC064, Newport, Irvine, CA, USA) on to a CMOS camera (GS3-U3-32S4M-C, FLIR, Wilsonville, OR, USA). Residual excitation light was filtered out by the dichroic filter and a short pass filter (ET680SP-2PS, Chroma, Bellows Falls, VT, USA). 

For all experiments, fluorescence signals were collected in epi-fluorescence mode. Data from the camera were transferred using control software written using LabVIEW 2015 (National Instruments, Austin, TX, USA), which also controlled an objective piezo positioner (MIPOS-500, Piezosystem Jena, Jena, Germany) in order to obtain focal stacks.

Because a DMD can also act as a diffraction grating, there exists a remote possibility that this dispersion will affect the system. The effect of the DMD’s dispersion is unlikely to have a major effect, since the “grating period” of the DMD in our configuration is high compared to that of the grating. Nevertheless, since the DMD is imaged onto the sample through an achromatic lens system, any additional dispersion from the DMD is actually likely to *improve* the axial resolution slightly, since this dispersion will lead to additional separation of the spectral components at the back aperture of the microscope objective.

### 2.2. Preparation of Calibration and Biological Samples

A thin, fluorescent layer of green quantum dots (supplied by QDVision, Lexington, MA, USA) was used to characterize the axial resolution of the system. Quantum dots dispersed in hexane (10 µL) were dropped onto a coverslip (thickness 170 µm) and allowed to dry. The coverslip was affixed to a glass slide and sealed. Another, more disperse quantum dot sample was prepared by diluting the quantum dot solution by a factor of 400 and repeating the procedure. A pollen grain slide (Carolina Biological Supply Inc., Burlington, NC, USA) was used to visually compare the optical sectioning capability of wide-field vs. line scanning two-photon temporal focusing. We further used a prepared slide of sectioned mouse kidney (F24630, Invitrogen, Carlsbad, CA, USA) to demonstrate the utility of DMD-based line scanning two-photon temporal focusing. For all line scanning measurements, each line pattern was illuminated for 2 ms, and total imaging time per frame was 1–2 s. For the line scanning modes, the generated lines swept the imaged area multiple times per frame. For example, a 1-pixel-thick line would sweep an area 128 pixels thick in 256 ms. Therefore, for an integration time of two seconds, the entire area was swept approximately eight times. For wide-field mode, square patterns were illuminated for less than 40 ms per frame. Excitation powers were adjusted to avoid saturation of the camera. 

## 3. Results and Discussion

### 3.1. Axial Resolution Comparison Between Wide-Field and Line-Scanning Temporal Focusing

A thin layer of green fluorescent quantum dots (QD) was used to compare the axial resolution of wide-field temporal focusing and DMD-based line scanning temporal focusing techniques. Four measurements were performed for each imaging mode, and the integrated fluorescence intensity profile in the *z*-axis (*I*_p,z_) was fitted using a Lorentzian function. The full-width half maximum (FWHM) of the fitted Lorentzian functions were averaged and used as a quantitative indicator of the axial resolution throughout this work. 

For the wide-field temporal focusing, 128 × 128 pixels at the center of the DMD were set to an “on” state during the illumination time. The rest of the micromirrors outside the selected area remained “off” at all times. A line pattern was generated using 128 × 3 “on” micromirror pixels, and subsequent lines of the same thickness were scanned on the DMD to sweep the same area as the 128 × 128 pixel wide-field mode. Excitation powers were adjusted so as not to saturate the fluorescence signals from the thin quantum dot layer. 

**Figure 2:**
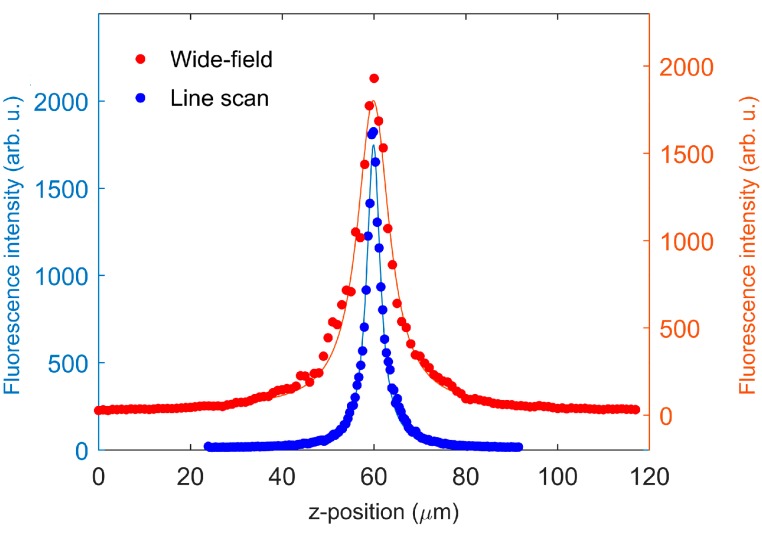
The comparison of *z*-axis integrated fluorescence intensity profile from wide-field temporal focusing excitation (red dots) and line scan temporal focusing excitation (blue dots). Pixels of the DMD with dimensions of 128 × 128 were “on” for the wide-field measurement, and a sequence of 128 × 3 pixels were “on” for the line scan mode. The data were fitted with Lorentzian function (solid lines).

Wide-field (128 × 128 “on” pixels) illumination resulted in an axial FWHM of 9.03 ± 0.38 µm (mean ± SEM, *N* = 4) from *I*_p,z_ (Figure 2). This value is close to the FWHM 7.6 µm obtained from our previous work (the literature value was obtained with an oil immersion 40×/1.3 NA objective) [17] but slightly broader, possibly due to underfilling of the back aperture of the objective. Although the initial microscope design deliberately did not overfill the back aperture of the objective lens in order to maximize illumination intensity at the sample plane, this reduction in axial resolution relative to the literature should affect both the wide-field and line-scanning modalities equally; hence, relative comparisons of the two different modalities is still valid. The FWHM of the line pattern illumination (128 × 3 “on” pixels) was determined to be 3.55 ± 0.02 µm, which is significantly reduced compared to the wide-field illumination. This result clearly demonstrates the improved axial resolution of line scanning over that of wide-field illumination. 

### 3.2. Axial Resolution Dependence on the Line Thickness and the Number of Lines

**Figure 3:**
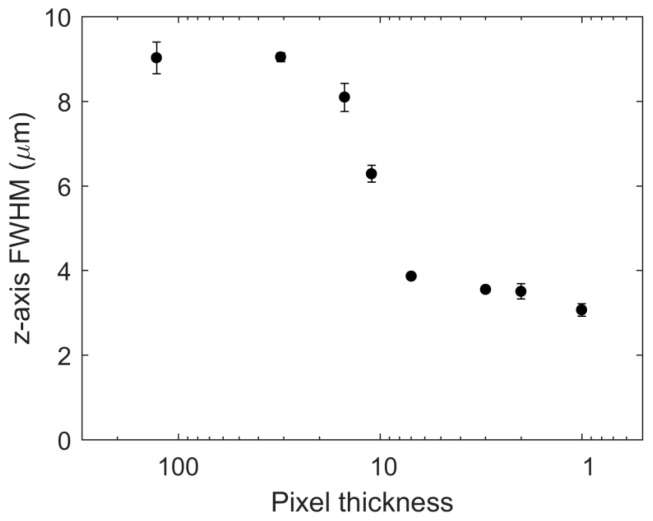
The dependence of *I*_p,z_ full-width half maximum (FWHM) of a thin quantum dot layer upon illumination with pixel thicknesses of line (128 × *T*) excitation, generated by DMD. Four measurements (*N* = 4) were performed for each pixel thickness and fitted with Lorentzian function. Pixel thicknesses corresponded to *T* = 128, 31, 15, 11, 7, 3, 2, and 1.

We further studied the effect of pixel thickness on *I*_p,z_ FWHM. The thickness of the lines (each of which had a length of 128 pixels at the DMD) was changed from 128 (wide-field), to 31, 15, 11, 7, 3, 2, and 1 pixels. As an illustration of the scanning process, a line of thickness of 1 was composed of 128 sequentially projected patterns of 128 × 1 “on” pixels, in order to illuminate the same area as the 128 × 128 wide-field illumination. Individual line patterns were illuminated for 2 ms and camera exposure times were kept to 1–2 s to maximize the image signal-to-noise ratio. For each pixel thickness, four *z*-stacks were obtained. *I*_p,z_ plots were obtained from each *z*-stack measurement and fitted with a Lorentzian function. The averaged Lorentzian fit result is summarized in Figure 3 and Table 1. Interestingly, the *I*_p,z_ FWHM exhibited a threshold-like response, rapidly transitioning from a wide-field like FWHM to a single-pixel line-like FHWM with a line thickness of 11 pixels, which corresponds to a thickness of ~106 µm immediately after the DMD, or ~1.17 µm at the sample once it has been imaged by the tube lens (*f* = 750 mm) and the objective lens (*f*_eff_ = 8.25 mm). The FWHM of *I*_p,z_ continued to drop as the line thickness decreased, but not to as great a degree. The minimum FWHM 3.07 ± 0.15 µm was obtained when the line was 1 pixel thick. This FWHM (3.07 µm) is significantly reduced compared to that (9.03 µm) of the wide-field illumination of the same illumination width. However, it is still broader than the theoretical diffraction limit of point scanning, which is ~1.1 µm ($\left(0.886\lambda /\sqrt{2}\right)\left(1/(n-\sqrt{n^{2}-NA^{2}})\right)$, where λ = 800 nm, *n* = 1.3, and *NA* = 1.0, equivalent to our experimental conditions) [20,21], as well as the FWHM (~1.5 µm) of line temporal focusing achieved using a cylindrical lens [18]. The broader FHWM is possibly due to the underfilled back aperture of the objective as mentioned above. To exclude the possibility of a thick QD layer broadening the FWHM, we prepared another QD slide after diluting the QD solution by a factor of 400, and observed a similar *I*_p,z_ FWHM of 3.57 ± 0.20 µm from 3-pixel-thick line illumination, confirming that the QD slide that was used was sufficiently thin. 

To explain the threshold-like response, we propose a “two-regime” model in which the axial resolution is constrained by two different phenomena—the temporal focusing and the geometric focusing effects. In the first regime, with a wide thickness, the dominant effect is temporal focusing—out-of-plane excitation is suppressed by the temporal focusing dispersion, so no matter how wide the excitation, there is a limiting axial resolution. As the line thickness becomes thinner, geometric focusing also contributes—as one focuses the line tighter and tighter, axial resolution is improved in exactly the same way as a normal multiphoton microscope. Finally, the resolution plateaus as the diffraction limit and residual optical aberrations in the system start to constrain how narrow the projected lines can be.

The most salient benefit of using a DMD-based microscope is to generate arbitrarily patterned illumination; multiple lines of various thicknesses can be generated by a DMD, while conventional line temporal focusing using a cylindrical lens [18] can only produce one. We generated multiple 3-pixel-thick lines with the DMD and measured *I*_p,z_ FWHM of the thin QD slide (Table 2). FWHMs of *I*_p,z_ remained in the region of 3.4–3.5 µm, from a single line up to 16 lines (duty cycles, or the ratio of on and off pixels, were 0.023, 0.094, 0.19, and 0.38 for 1, 4, 8, and 16 lines, respectively); thus, each individual line behaved in an isolated manner, not affecting the other projected lines. With 32 lines, however, corresponding to duty cycle of 0.73, the FWHM of 7.75 µm became worse by a factor of 2 compared to 1, 4, 8, and 16 lines. The upper limit of multiple lines illumination is wide-field illumination, in which the FWHM is 9.03 µm (Table 1). Knowing the limit of the number of lines that can be exposed while maintaining a minimum level of axial resolution is important to maximize imaging speed; the exposure time is inversely proportional to the number of identical lines. As a guide, illuminating multiple lines with a duty cycle below ~0.4 is a safe estimate in order to reduce the image acquisition time while maintaining the axial resolution of the system.

### 3.3. Imaging of a Pollen Grain and Biological Sample

**Figure 4:**
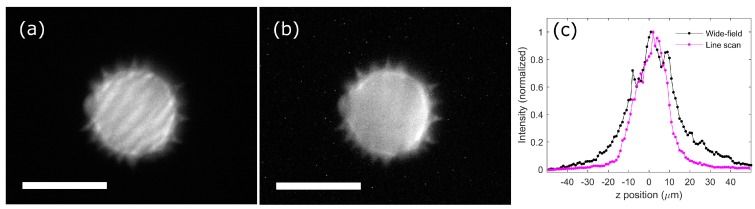
Two-photon temporal focusing fluorescence images of a pollen grain. The pollen grain was imaged with wide-field, 128 × 128 “on” pixels and 8 lines of 128 × 3 “on” pixels. The images of the pollen grain at maximum fluorescence intensity by the wide-field and the line scan modes are shown (**a**,**b**), respectively. (**c**) Normalized fluorescence *z*-profile of the wide-field (black) and the line scan (magenta) modes on the pollen grain. Scale bar is 30 µm in (**a**,**b**).

We collected two-photon temporal focusing fluorescence images of a pollen grain in order to demonstrate the utility of DMD-based line temporal focusing (Figure 4). The pollen grain was imaged at 1 µm steps along the *z*-axis, both with equally spaced 8 lines (128 × 3 “on” pixels) and with wide-field (128 × 128 “on” pixels) temporal focusing. Clearly, DMD-generated line scanning displayed a sharper axial fluorescence intensity profile than wide-field illumination on the same pollen grain (Figure 4c). In addition to better axial optical sectioning, line scanning removes the “ghost” interference pattern that appears in the wide-field mode (Figure 4a); the striped pattern on the pollen grain is not a real feature. The ghost pattern potentially originates from interference among multiple spectral components or between multiply reflected beams passing through the many optical components in the system.

**Figure 5:**
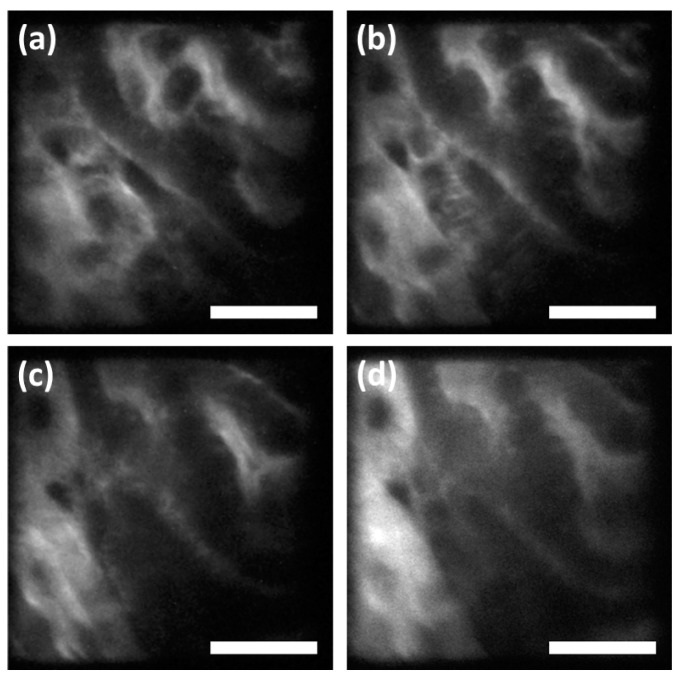
Line scanning temporal focusing two-photon excited fluorescence images of an excised mouse kidney slice. Three of the line scanning (16 lines of 256 × 3 “on” pixels) *z*-stack images taken at 2 µm intervals are shown in (**a**–**c**). Scale bar; 20 µm. Wide-field (256 × 256 “on” pixels) temporal focusing image at the same *z*-position with (**b**) is shown for comparison in (**d**).

We next imaged a prepared slide of sectioned mouse kidney (F24630, Invitrogen). The sample was imaged at a 0.5 µm step size with a 40 µm axial scanning range to ensure that no sample features were missed. Both line scanning (16 lines of 256 × 3 “on” pixels corresponding to duty cycle of 0.19) and wide-field (256 × 256 “on” pixels) illumination were used. While convoluted tubule structures in a mouse kidney can be identified by both the line scanning (Figure 5a–c) and wide-field (Figure 5d) temporal focusing, fine structures are more discernible by line scanning mode. 

## 4. Conclusions

We have demonstrated a line-scanning temporal focusing two-photon excited fluorescence microscope using a digital micromirror device. While wide-field temporal focusing can only achieve a broad axial resolution of 9.03 µm measured using a thin fluorescent quantum dot layer, line-scanning temporal focusing generated by a DMD shows an enhanced axial resolution of up to 3.07 µm, which is an improvement of a factor of three over that of the wide-field mode. We were also able to determine that, by illuminating the sample with lines of different thicknesses, the transition between wide-field-like and single-line-like axial resolutions occurs very rapidly. This observation leads us to recommend the use of multi-pixel thick line scans for a better signal-to-noise ratio without sacrificing axial resolution. Equally spaced multiple lines were also examined, confirming that multiple lines do not degrade the axial resolution until a certain duty cycle is reached, which was ~0.4 for our experimental conditions. 

We also have visually compared the axial resolution by wide-field and multiple line scanning temporal focusing microscopy of the autofluorescence of a pollen grain. The fluorescence trace from the pollen grain as a function of focal position clearly demonstrated that line scanning temporal focusing has better optical sectioning capability than the wide-field mode. We also observed that the line scanning mode can remove interference patterns that appeared in the wide-field mode; patterns which might mislead the user under some circumstances. A mouse kidney slice was imaged using the line-scanning mode, which produced a sharper transition from the background to fluorescence signal at the boundary between the kidney tissue and the coverslip. This result demonstrates the utility of DMD-based line scanning temporal focusing two-photon excited fluorescence in the field of biomedical imaging. 

## Figures and Tables

**Table 1:** FWHM of a thin quantum dot slide varying the thickness of line pattern on the DMD. Four measurements (*N* = 4) were performed and averaged for each pixel thickness (pixel size of 128 × *T*).

Pixel Thickness (128 × *T*)	FWHM (µm)	Standard Error (µm)
128 (wide-field)	9.03	0.38
31	9.04	0.10
15	8.10	0.33
11	6.29	0.20
7	3.87	0.05
3	3.55	0.02
2	3.51	0.18
1	3.07	0.15

**Table 2:** Axial FWHMs of a thin quantum dot layer, varying the number of illuminating lines when single or multiple 128 × 3-pixel-thick lines were used. Line patterns were spaced equally so that the total number of patterns decreased as the number of lines increased, in order to illuminate the same sample area, corresponding to 128 × 128 “on” pixels. Four measurements (*N* = 4) were performed, and fitted FHWMs were averaged for each pixel thickness.

Number of Lines (*L* × (128 × 3))	FWHM (µm)	Standard Error (µm)
1	3.55	0.02
4	3.40	0.12
8	3.38	0.17
16	3.40	0.23
32	7.75	0.43
